# The Association Between Syphilis Infection and HIV Acquisition and HIV Disease Progression in Sub-Saharan Africa

**DOI:** 10.3390/tropicalmed10030065

**Published:** 2025-02-28

**Authors:** Sindhuri Gandla, Raja Nakka, Ruhul Ali Khan, Fatemeh Salboukh, Musie Ghebremichael

**Affiliations:** 1 Ragon Institute of MGH, MIT, and Harvard, Cambridge, MA 02139, USA; sgandla@mgh.harvard.edu (S.G.); rnakka@mgh.harvard.edu (R.N.); 2 Department of Mathematics, University of Arizona, Tucson, AZ 85721, USA; ruhul@arizona.edu; 3 Department of Engineering and Applied Science, University of Massachusetts, Dartmouth, MA 02747, USA; fsalboukh@umassd.edu; 4 Harvard Medical School, Massachusetts General Hospital, Cambridge, MA 02115, USA

**Keywords:** sub-Saharan Africa, HIV, syphilis, HIV acquisition, HIV progression, logistic regression

## Abstract

Syphilis and other sexually transmitted infections (STIs) are highly prevalent in most regions experiencing severe human immunodeficiency virus (HIV) epidemics. In sub-Saharan Africa, the region most heavily affected by HIV, the prevalence of syphilis among people living with HIV (PLWH) is notably high. This region accounts for 40% of global STIs and 70% of HIV cases. Despite the high prevalence of syphilis and other STIs among PLWH in the region, there are limited studies on the interplay between the two infections from the region. Most studies on the association between syphilis and HIV transmission/progression from the region are limited to specific groups of people, such as female sex workers or pregnant women. In this manuscript, we evaluated the association between the two infections using population-based surveys conducted in the region. Statistical methods (such as logistic regression models and propensity score matching) were employed to assess the interplay between the two infections. Our findings indicated that syphilis infection was associated with higher odds of HIV acquisition. Moreover, co-infection with syphilis was associated with higher odds of HIV disease progression among antiretroviral therapy (ART)-treated PLWH, though the association did not reach statistical significance. Our findings suggest that the recognition and treatment of syphilis to reduce the risk of HIV acquisition/progression should be a public health priority in sub-Saharan Africa, where ART may not be readily available.

## 1. Introduction

Syphilis is still a modern-day pandemic that affects millions of individuals globally and is becoming more common [1]. It is caused by Treponema pallidum and mainly spreads through sexual activity [1,2,3]. The infection progresses through four stages (primary, secondary, latent, and tertiary), with signs and symptoms varying at each stage [2,3]. If left untreated, syphilis can impact multiple organs over the years or even decades as it advances through its various stages [1,2,3]. In 2022, the World Health Organization (WHO) projected that 8 million people aged 15 to 49 contracted syphilis globally, with an additional 700,000 cases of congenital syphilis [4,5]. New syphilis cases among adults aged 15 to 49 rose from 7.1 million in 2020 to 8.0 million in 2022 [6]. The global prevalence rate of syphilis was reported to be 0.6% (95% CI, 0.5–0.7%) in both men and women, and congenital syphilis at a rate of 523/100,000 live births [3,4,5]. To curb syphilis prevalence, the WHO has set two goals for 2030: (1) reduce the global incidence of syphilis by 90% and (2) decrease the rate of congenital syphilis to ≤50 cases per 100,000 live births [4,7]. While penicillin is the conventional treatment for syphilis, challenges such as the need for injections, medication shortages, and allergies remain barriers to effective treatment [1,2,3].

Syphilis is known to increase the risk of HIV transmission [8,9,10]. The presence of syphilitic sores or ulcers, particularly in genital or anal areas, creates entry points for HIV, facilitating easier transmission during unprotected sexual contact [3]. For this reason, individuals with syphilis have a higher risk of acquiring HIV [3]. Additionally, both HIV and syphilis have shared risk factors like multiple sexual partners, thereby facilitating co-infections [11,12,13]. Thus, routine syphilis screening and treatment play a key role in reducing HIV incidence [11]. Preventative strategies like doxycycline postexposure prophylaxis (doxy-PEP), which refers to taking the antibiotic doxycycline after exposure to STIs, have shown promise in reducing syphilis incidence in high-risk populations [14,15]. However, HIV complicates syphilis treatment efficacy, with several studies reporting serological failure even after appropriate syphilis treatment [8,9,10,14,16].

Syphilis can also compromise HIV treatment efficacy in co-infected individuals. Syphilis and other sexually transmitted infections (STIs) worldwide are highly prevalent among people living with HIV (PLWH) [1,2,17]. A recent study from sub-Saharan Africa found that syphilis prevalence among PLWH in the region was approximately 7.3% (95% CI, 6.3–8.5%) [17]. Antiretroviral therapy (ART) has proven to be highly effective for HIV disease treatment, leading to viral load suppression. Early ART initiation and adherence to ART reduce disease progression and death while also preventing new cases from spreading [18]. However, PLWH experience a higher risk of recurrent syphilis, thereby affecting ART efficacy [19]. Studies have shown that syphilis can persist even in PLWH with suppressed viral loads and some individuals on ART still experience virological failure due to syphilis [20,21,22]. Besides virological failure, studies have also shown that untreated syphilis in co-infected individuals can lead to substantial neurologic and cardiovascular consequences as well as significant morbidity and mortality [3,17]. These findings highlight the critical role of syphilis screening and treatment in improving ART efficacy and reducing HIV disease progression. Though several studies have explored the role of ART in achieving VL suppression, the association between syphilis and HIV acquisition/disease progression is not well studied, especially in regions where treatment is not readily available. In this manuscript, we perform a secondary data analysis to evaluate the association between syphilis and HIV acquisition/progression. We used data from large population-based surveys conducted in sub-Saharan Africa, as the region bears 40% of the global burden of STIs and 70% of the global HIV burden [6].

## 2. Materials and Methods

**Study Design and Participants:** In this study, we performed a secondary analysis of retrospective cross-sectional data from 53,915 adult participants enrolled through the Population-based HIV Impact Assessment (PHIA) surveys conducted in Zambia (*n* = 21,280) and Tanzania (*n* = 32,635). The PHIA project was funded by the U.S. President’s Emergency Plan for AIDS Relief (PEPFAR) and technically supported by the International Center for AIDS Care and Treatment Programs (ICAP) at Columbia University through the U.S. Centers for Disease Control and Prevention (CDC) [23,24,25,26]. The survey questionnaire and consent forms were translated from English into local languages. An electronic informed consent form was completed on a tablet, where participants gave verbal consent that was recorded directly on the device. The survey included three stages: head-of-household interview, individual interview, and biomarker testing. The household questionnaire collected information about household residents. The individual adult questionnaire covered topics such as HIV treatment and care, male circumcision, and HIV knowledge. It also included questions about behavioral risk factors related to HIV, as well as syphilis, tuberculosis, and cervical cancer. Eligible participants aged 15 and older completed the standardized questionnaire and provided blood samples for HIV and syphilis testing [26,27]. The survey followed each country’s national guidelines, and HIV home-based testing and counseling (HBTC) were conducted. The screening test was conducted using a sequential rapid-test algorithm that includes the Determine HIV-1/2 test (Abbott Molecular Inc., Des Plaines, IL, USA) and the Uni-Gold test (Trinity Biotech, Wicklow, Ireland) as the confirmatory test. Syphilis testing was conducted using Chembio DPP Syphilis Screen & Confirm Assay (Chembio Diagnostic Systems, Inc., Medford, NY, USA) to detect antibodies against treponemal and non-treponemal antigens. Active syphilis infection is defined as the presence of both treponemal and non-treponemal antibodies, whereas a history of syphilis is defined as the presence of only treponemal antibodies. The entire sample size (*n* = 53,915) was used to analyze the association between syphilis and HIV acquisition. For the analysis of HIV disease progression, we applied propensity score matching (PSM) to match HIV-positive participants co-infected with syphilis with those without syphilis based on age and gender. After matching, the final sample included 792 participants in Zambia and 542 participants in Tanzania, ensuring comparable groups for analysis.

All PHIA survey materials, consent forms, screening forms, referral forms, recruitment materials, and questionnaires were reviewed and approved by the relevant ethics and regulatory bodies in the country, as well as by the institutional review boards of Columbia University Medical Center, Westat, and the U.S. Centers for Disease Control and Prevention. External monitoring took place twice during each survey. The survey management teams monitored any adverse events or protocol deviations, which were promptly reported to the institutional review boards. The detailed methodology, including survey questionnaires, study design, and testing procedures, was described previously [26,27,28].

**Study Outcome and Covariates:** The primary outcomes of interest in this paper were HIV acquisition and HIV disease progression. HIV disease progression was assessed among the ART-treated PLWH by lack of HIV RNA VL suppression, defined as ≥1000 copies/mL. The covariates we considered include socio-demographic factors (such as age and gender), STI (co-infection) status, and clinical variables (such as duration on ART and CD4 T cell count).

**Statistical Analysis:** Descriptive measures and statistical graphs were used to summarize data. Continuous variables such as age and CD4 T cell count were summarized using medians and interquartile ranges (IQRs), while categorical variables were presented as frequencies and percentages. Categorical variables were compared between groups using Fisher’s exact test, whereas the non-parametric Wilcoxon rank-sum test was used to compare continuous study variables. The main objectives of our analyses were twofold: the first objective was to evaluate the association between syphilis and HIV disease acquisition. Logistic regression models were used to assess the association between syphilis and HIV acquisition after adjusting for the effect of other factors such as age and gender. The second objective was to evaluate the association between syphilis and HIV disease progression in ART-treated PLWH. HIV disease progression was defined by the failure of HIV RNA viral load (VL) suppression. We used propensity score matching (PSM) to reduce potential confounding and ascertain the independent association between syphilis and HIV disease progression. HIV patients co-infected with syphilis were matched for age and gender with HIV patients without syphilis co-infection. The matching was performed in R (version 4.4.2) using the MatchIt package (version 4.6.0) [29]. HIV RNA VL suppression rates were analyzed by comparing participants co-infected with syphilis with age–gender-matched HIV participants without syphilis co-infection. Logistic regression models were used to assess the association between syphilis and lack of VL suppression after adjusting for the effect of other confounding variables such as CD4 T cell count, duration on ART treatment, and ART initiation 12 months or more before the survey. Statistical analyses were conducted using R (version 4.4.2) [30]. All *p*-values are two-sided, and a *p*-value of less than 0.05 was considered statistically significant.

## 3. Results

We analyzed data from 21,280 participants in Zambia and 32,635 participants in Tanzania who took part in the surveys. Participants’ ages ranged from 15 to 59 years in Zambia and 15 to 64 years in Tanzania. Table 1 provides an overview of the participant characteristics. The median age of the participants was 29 (IQR = 21–39) years in Zambia and 31 (IQR = 22–45) years in Tanzania. In both countries, female participation was higher than male, with females making up 57% (*n* = 12,109) in Zambia and 56% (*n* = 18,172) in Tanzania. A total of 19,157 (90%) participants from Zambia and 32,634 (99%) from Tanzania were tested for syphilis. Of those tested, 2.8% (*n* = 591) in Zambia and 1% (*n* = 328) in Tanzania tested positive for active syphilis. More than 6% of participants in both countries had a history of syphilis, with 6.4% (*n* = 1361) in Zambia and 6.2% (*n* = 2026) in Tanzania. A total of 19,115 participants in Zambia and all participants in Tanzania were screened for HIV. Among those screened, 11.6% (*n* = 2467) in Zambia and 5.8% (*n* = 1895) in Tanzania tested positive for HIV. Of those who tested positive for HIV and started ART, 7% (*n* = 1465) in Zambia and 3% (*n* = 994) in Tanzania achieved VL suppression.

Table 2 outlines the characteristics of the study participants by HIV acquisition status in Zambia and Tanzania. Among those screened for HIV, 13% (*n* = 2467) in Zambia and 6% (*n* = 1895) in Tanzania tested positive for HIV. The median age of the participants was notably higher among PLWH, with a median age of 38 years (IQR = 30–45) in Zambia and 39 years (IQR: 30–47) in Tanzania, suggesting that older individuals were more susceptible to acquiring HIV (*p* < 0.001). In both countries, most of the PLWH were females: 69% (*n* = 1688) in Zambia and 69% (*n* = 1297) in Tanzania. Moreover, participants with active syphilis or a history of syphilis were found to be at increased risk of HIV acquisition. Among participants with active syphilis, 9.1% (*n* = 225) tested positive for HIV in Zambia. Similarly, in Tanzania, among the participants with active syphilis, 4.0% (*n* = 76) tested positive for HIV. As with the case of active syphilis, a history of syphilis was also significantly (*p* < 0.001) associated with an increased risk of HIV acquisition in both countries. In Zambia, among the participants with a history of syphilis infection, 16.4% (*n* = 406) were HIV positive. Similarly, in Tanzania, among the participants having a history of syphilis, 14.3% (*n* = 271) tested as HIV positive. In conclusion, our analysis indicates that older age, female gender, and active syphilis infection or history of syphilis were associated with an increased risk of HIV acquisition.

In both countries, less than 20% of the PLWH were co-infected with syphilis. For this reason, we used propensity score matching (PSM) to select age–gender-matched PLWH without syphilis for each co-infected subject. Table 3 displays the patient characteristics by HIV VL suppression status for co-infected PLWH and the matched PLWH without syphilis infection. Among PLWH, 61.5% (*n* = 487) in Zambia and 55% (*n* = 299) in Tanzania achieved VL suppression. In both countries, the proportion of PLWH who achieved VL suppression was lower in those with active syphilis: Zambia (26.1%, *n* = 127) and Tanzania (12.0%, *n* = 36), and the difference was not statistically significant (*p* = 0.123 in Zambia and *p* = 0.171 in Tanzania). In Zambia, PLWH with a history of syphilis achieved less VL suppression (49.7%, *n* = 242), whereas in Tanzania, the proportion of PLWH with a history of syphilis achieved higher VL suppression rates (52.5%, *n* = 157). This difference was also not statistically significant (*p* = 0.715 in Zambia and *p* = 0.226 in Tanzania). Table 3 also compares other covariates by suppression status. In both countries, PLWH who achieved VL suppression were older: Zambia (median = 40 years, IQR = 32–46) and Tanzania (median = 43 years, IQR: 36–52), when compared to those without suppression, which was statistically significant (*p* < 0.001). In both countries, participants with VL suppression had higher CD4 T cell counts: Zambia (median = 475 cells/µL, IQR: 344–635) and Tanzania (median = 483.5 cells/µL, IQR: 346.8–655) compared to those without suppression, which was statistically significant (*p* < 0.001). Although a higher proportion of females achieved VL suppression in both countries, gender was not statistically significant (*p* = 0.07 in Zambia and *p* = 0.06 in Tanzania). In both countries, participants taking ART medication achieved higher VL suppression rates, with 93.8% (n = 457) in Zambia and 88.6% (n = 265) in Tanzania, and it was statistically significant (*p* < 0.001). In Zambia, a lower proportion of PLWH who were not on ART achieved VL suppression (14.2%, *n* = 69). Similarly, 11% (*n* = 33) of PLWH who were not on ART achieved VL suppression in Tanzania. Whereas, in Zambia, PLWH who are on ART treatment for 24 months or more achieved higher VL suppression (57.3%, *n* = 279) when compared to PLWH on ART treatment for 12–23 months (11.5%, *n* = 56) and less than 12 months (13.5%, *n* = 66). Similarly, in Tanzania, higher VL suppression was achieved among PLWH and on ART treatment for 24 months or more (62.2%, *n* = 186) when compared to PLWH on ART treatment for 12–23 months (9%, *n* = 27) and less than 12 months (0%). ART duration was statistically significant in achieving VL suppression (*p* < 0.001) in both countries.

**Figure 1:**
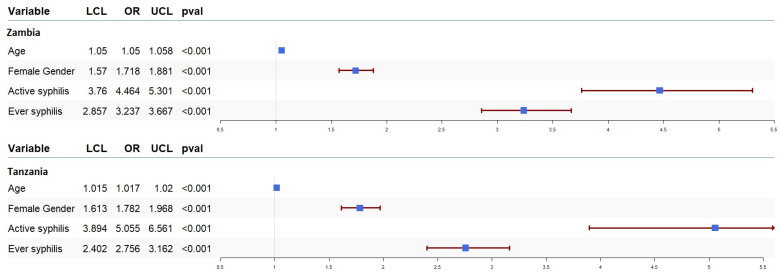
Forest plot of odds ratios (OR) together with 95% confidence intervals for HIV acquisition in Zambia and Tanzania. The OR is shown by the blue box, and horizontal lines show the confidence interval (CI). The arrow mark denotes the expanded CI beyond the x-axis limitations, while the ends of the lines represent the CI’s borders.

We ran univariate and multivariate logistic regression to assess the effect of syphilis on the odds of HIV acquisition and progression. The results of our univariate analysis for HIV acquisition are presented in Figure 1. In our sample data from both countries, syphilis infection was associated with higher odds of HIV acquisition. The odds of HIV acquisition in participants with active syphilis infection in Zambia and Tanzania were OR = 4.46 (95% CI: 3.76–5.30, *p* < 0.001) and OR = 5.05 (95% CI: 3.89–6.56, *p* < 0.001), respectively. Moreover, the odds of HIV acquisition in participants with a history of syphilis in Zambia and Tanzania were OR = 3.23 (95% CI: 2.85–3.66, *p* < 0.001) and OR = 2.75 (95% CI: 2.40–3.16, *p* < 0.001), respectively. We also ran multivariate logistic regression to estimate the association between syphilis and HIV acquisition after adjusting for the effect of other covariates, including gender and age. These results also showed that both active syphilis (Zambia (OR = 2.37, 95% CI: 1.86–3.02, *p* < 0.001) and Tanzania (OR = 2.58, 95% CI: 1.91–3.48, *p* < 0.001)) and a history of syphilis infection (Zambia (OR = 1.77, 95% CI: 1.48–2.12, *p* < 0.001) and Tanzania (OR = 1.94, 95% CI: 1.65–2.29, *p* < 0.001)) were associated with higher odds of HIV acquisition regardless of the other covariates.

**Figure 2:**
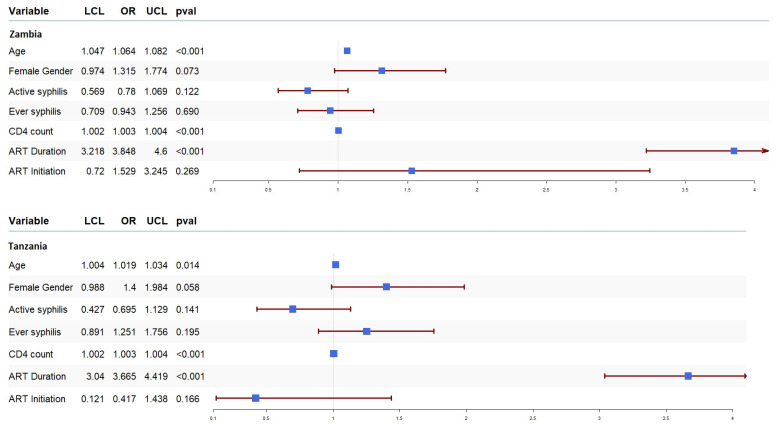
Forest plot of odds ratios (OR) together with 95% confidence intervals for HIV progression among PLWH in Zambia and Tanzania. The OR is shown by the blue box, and horizontal lines show the confidence interval (CI). The arrow mark denotes the expanded CI beyond the x-axis limitations, while the ends of the lines represent the CI’s borders.

To understand the association between syphilis and HIV progression, we ran a univariate logistic regression, and the results are presented in Figure 2. In both countries, active syphilis was associated with a lack of VL suppression, though it did not reach statistical significance: Zambia (OR = 0.78, 95% CI: 0.56–1.06, *p* = 0.1225) and Tanzania (OR = 0.695, 95% CI: 0.427–1.129, *p* = 0.141). Similarly, a history of syphilis was associated with a lack of viral suppression in Zambia (OR = 0.94, 95% CI: 0.70–1.25, *p* = 0.690) but inclined towards suppression in Tanzania (OR = 1.25, 95% CI: 0.89–1.75, *p* = 0.195). Figure 2 also displays the effect of the other covariates on VL suppression. In both countries, ART duration (Zambia (OR = 3.84, 95% CI: 3.21–4.60, *p* < 0.001) and Tanzania (OR = 3.66, 95% CI: 3.04–4.41, *p* < 0.001)) and CD4 count (Zambia (OR = 1.003, 95% CI: 1.002–1.004, *p* < 0.001) and Tanzania (OR = 1.003, 95% CI: 1.002–1.004, *p* < 0.001)) were associated with VL suppression. Similarly, older age in Zambia (OR = 1.064, 95% CI: 1.04–1.08, *p* < 0.001) was also associated with VL suppression.

## 4. Discussion

The major focus of our paper was twofold: to assess the association between syphilis and HIV acquisition, as well as the association between syphilis and HIV disease progression in sub-Saharan Africa. We used retrospective data from 53,915 adult participants enrolled through population-based studies in Zambia and Tanzania. In our Zambia data, 11.6% of the participants were PLWH, while in our Tanzania data, 5.9% of the study participants were PLWH. Among the PLWH, the proportion of participants with either active syphilis infection or a history of syphilis infection was 25.5% (*n* = 631) in Zambia and 18.3% (*n* = 347) in Tanzania. These rates exceeded those reported in previous studies conducted across sub-Saharan Africa [31,32]. In both countries, people with active syphilis are four times more likely to have HIV infection, and a history of syphilis was associated with more than a twofold increase in HIV acquisition, which is consistent with previous studies in sub-Saharan Africa [12,27,31,33]. Moreover, our findings also indicate that older adults and females were at an increased risk of acquiring HIV in both countries, consistent with previous research from the region [12,34].

The second objective of our manuscript was to evaluate the association between syphilis and HIV disease progression in ART-treated PLWH. Our analyses revealed that, in both countries, active syphilis or a history of syphilis was associated with a lack of VL suppression, though it did not reach statistical significance. This limits our ability to draw definitive conclusions regarding the impact of syphilis infection on HIV disease progression. One possible explanation is ART adherence, which may have played a role in maintaining viral suppression despite syphilis co-infection. A study in China found that individuals with HIV/syphilis co-infection who maintained sustained ART adherence exhibited similar viral suppression and immune response to those without syphilis [35]. Another study reported that viral suppression rates remained stable before, during, and after syphilis infection, suggesting that syphilis did not compromise viral suppression in individuals adhering to ART [36]. Given these findings, it is possible that factors like ART adherence and syphilis treatment may have influenced the observed relationship in our study population. 

Our study has many advantages over previous related studies. We used a large population-based study, which reduces the potential biases associated with small sample sizes or specific groups of participants like men who have sex with men (MSM), female sex workers, or pregnant women. Thus, the results of our analysis provide valuable insights that can help policymakers understand the effects of co-infections, like syphilis, on HIV acquisition and progression. The higher odds of HIV acquisition among syphilis-co-infected patients emphasize the importance of integrating syphilis diagnosis and treatment services in HIV prevention programs. This integration should also consider the cost implications and the financial burden on healthcare systems, including diagnostic tools, personnel, and infrastructure in resource-limited settings. However, our findings should be interpreted with caution due to some limitations. One major disadvantage is the use of retrospective cross-sectional data, which might not consider changes over time in essential variables like healthcare access or high-risk behavioral characteristics. Additionally, our data cannot determine the sequence of infections (whether syphilis preceded HIV or vice versa) or whether participants received treatment for syphilis. Another limitation is the potential for inherent bias in the retrospective data, which affects our ability to assess the impact of co-infections on HIV progression fully. While data on ART duration were available, ART adherence, an important factor in understanding the association between syphilis and HIV disease progression, was not. This also limits the generalizability of our findings. We also acknowledge that the data used in this study were collected from 2016–2017, prior to the COVID-19 pandemic. Since then, factors such as healthcare access and STI epidemiology may have changed. Additionally, the pandemic may have disrupted STI testing, treatment services, and HIV care in sub-Saharan Africa. While our analysis of the cross-sectional data provides valuable insights into the association between syphilis and HIV acquisition/progression in a large population, more recent data would improve the generalizability of these findings. Future research should incorporate ART adherence data and adopt a prospective longitudinal design to assess better the impact of syphilis on HIV acquisition and progression.

## 5. Conclusions

We used retrospective cross-sectional data from Zambia and Tanzania and applied statistical methods (logistic regression models and PSM) to identify the association between syphilis and HIV acquisition/progression. Our findings indicated that syphilis infection was associated with higher odds of HIV acquisition. Moreover, co-infection with syphilis was associated with higher odds of HIV disease progression among ART-treated PLWH, though the association did not reach statistical significance. Our findings suggest that recognizing and treating syphilis should be a public health priority in sub-Saharan Africa, where ART may not be readily available. Integrating syphilis screening and treatment into HIV prevention programs while considering the cost implications and financial burden on health systems can enhance early detection and improve patient outcomes, thereby reducing HIV disease progression [34]. This approach can also strengthen public health responses to combat both HIV and syphilis.

## Figures and Tables

**Table 1:** Characteristics of participants in the survey in Zambia and Tanzania.

	Zambia (*n* = 21,280)	Tanzania (*n* = 32,635)
Variable	N (%)	N (%)
Age	29 (IQR: 21–39)	31 (IQR: 22–45)
Female gender	12,109 (56.9%)	18,172 (55.7%)
Male	9171 (43.1%)	14,463 (44.3%)
Active syphilis		
*Yes*	591 (2.8%)	328 (1%)
*No*	18,566 (87.2%)	32,306 (99%)
Ever syphilis		
*Yes*	1361 (6.4%)	2026 (6.2%)
*No*	17,796 (83.6%)	30,608 (93.8%)
HIV acquired	2467 (11.6%)	1895 (5.8%)
Viral load suppressed	1465 (6.9%)	994 (3%)

**Table 2:** Characteristics of participants by HIV acquisition status in Zambia and Tanzania.

	Zambia			Tanzania		
Variable	HIV Positive (*n* = 2467, 12.9%)	HIV Negative (*n* = 16,648, 87.1%)	*p*-Value	HIV Positive (*n* = 1895, 5.8%)	HIV Negative (*n* = 30,740, 94.2%)	*p*-Value
	N (%)	N (%)		N (%)	N (%)	
Age	38 (IQR: 30–45)	27 (IQR: 20–38)	<0.001	39 (IQR: 30–47)	30 (IQR: 21–44)	<0.001
Female gender	1688 (68.4%)	9285 (55.8%)	<0.001	1297 (68.4%)	16,875 (54.9%)	<0.001
Active syphilis						
*Yes*	225 (9.1%)	366 (2.2%)	<0.001	76 (4%)	252 (0.8%)	<0.001
*No*	2242 (90.9%)	16281 (97.8%)		1819 (96%)	30,487 (99.2%)	
Ever syphilis						
*Yes*	406 (16.4%)	955 (5.7%)	<0.001	271 (14.3%)	1755 (5.7%)	<0.001
*No*	2061 (83.6%)	15,692 (94.3%)		1624 (85.7%)	28,984 (94.3%)	

**Table 3:** Characteristics of participants by HIV progression status in Zambia and Tanzania.

	Zambia			Tanzania		
Variable	HIV Suppressed (*n* = 487, 61.5%)	HIV Not Suppressed (*n* = 305, 38.5%)	*p*-Value	HIV Suppressed (*n* = 299, 55.1%)	HIV Not Suppressed (*n* = 243, 44.8%)	*p*-Value
	N (%)	N (%)		N (%)	N (%)	
Age	40 (IQR: 32–46)	32 (IQR: 28–42)	<0.001	43 (IQR: 36–52)	40 (IQR: 32–49)	<0.001
CD4 count	475 (IQR: 344–635)	333 (IQR:207–492.5)	<0.001	483.5 (IQR: 346.8–655)	328 (IQR: 191–485)	<0.001
Female gender	332 (68.2%)	189 (62%)	0.07	196 (65.5%)	140 (57.6%)	0.06
Active syphilis						
*Yes*	127 (26.1%)	95 (31.1%)	0.1235	36 (12.0%)	40 (16.5%)	0.171
*No*	360 (73.9%)	210 (68.9%)		263 (88%)	203 (83.5%)	
Ever syphilis						
*Yes*	242 (49.7%)	156 (51.1%)	0.7153	157 (52.5%)	114 (46.9%)	0.2266
*No*	245 (50.3%)	149 (48.9%)		142 (47.5%)	129 (53.1%)	
ART status	457 (93.8%)	33 (10.8%)	<0.001	265 (88.6%)	33 (13.6%)	<0.001
ART initiation	343 (70.4%)	34 (11.1%)	0.29	169 (56.5%)	27 (11.1%)	0.219
ART duration						
*Not on ART*	69 (14.2%)	264 (86.5%)	<0.001	33 (11.0%)	199 (81.9%)	<0.001
*On ART < 12 months*	66 (13.5%)	10 (3.3%)		0 (0%)	0 (0%)	
*On ART 12–23 months*	56 (11.5%)	4 (1.3%)		27 (9%)	5 (2%)	
*On ART 24 months or more*	279 (57.3%)	26 (8.5%)		186 (62.2%)	25 (10.3%)	

## Data Availability

The dataset used in the manuscript is available from the Population-based HIV Impact Assessment (PHIA) Center: https://phia.icap.columbia.edu/ (accessed on 11 October 2024).
